# Editorial: The comorbid anxiety and depression disorder in patients with epilepsy: Diagnosis, prevention and treatment

**DOI:** 10.3389/fneur.2022.974462

**Published:** 2022-09-14

**Authors:** Zucai Xu, Xin Tian, Xuefeng Wang, Yangmei Chen, Qi Xu, Giovanni Assenza

**Affiliations:** ^1^Department of Neurology, Affiliated Hospital of Zunyi Medical University, Zunyi, China; ^2^Department of Neurology, First Affiliated Hospital of Chongqing Medical University, Chongqing, China; ^3^Department of Neurology, Second Affiliated Hospital of Chongqing Medical University, Chongqing, China; ^4^Chinese Academy of Medical Sciences and Peking Union Medical College, Beijing, China; ^5^Unit of Neurology, Neurophysiology and Neurobiology, Department of Medicine, Università Campus Bio-Medico di Roma, Rome, Italy

**Keywords:** epilepsy, anxiety, depression, diagnosis, treatment

Epilepsy is a common neurological disease, and patients with epilepsy are a huge group. Their quality of life is not only affected by various comorbidities that follow epilepsy, but also the risk of comorbidities such as anxiety and depression is several times higher than that of people without epilepsy, which makes the comorbidity of epilepsy more and more difficult to ignore. In order to further expand the understanding of epilepsy comorbidities and gain a deeper understanding of their pathogenesis, diagnosis, prevention, and treatment, we have collected 14 pieces of literature related to epilepsy comorbidities, hoping to provide more hints for future research in this direction.

People with epilepsy are more likely to feel anxious than the general population or people with other chronic diseases. Rocamora et al. analyzed 493 patients in their hospital who underwent long-term video EEG monitoring and found that anxiety and depression were more prevalent in female patients, Hospital Anxiety and Depression Scale for anxiety (HADS-A), and Beck Depression Inventory (BDI) > 13. The study revealed the factors affecting patients' quality of life in the epilepsy monitoring unit and pointed out that specific treatment methods should be adopted according to the patient's condition in clinical work.

Similarly, Forthoffer et al. argue that newly diagnosed epilepsy patients must be prospectively assessed and screened for anxiety and depression to determine whether anxiety and depression will have an impact on the future course of care.

Rauh et al. searched a series of epilepsy anxiety assessment tools through PubMed to understand how the previous literature assessed the anxiety of epilepsy patients and analyzed what aspects these self-assessment questionnaires covered. Overall, they found that various questionnaires were used for anxiety assessment of different patients, but there is still a lack of validated assessment tools that can broadly cover epilepsy-related anxiety phenomenology in patients with epilepsy.

However, how to avoid anxiety in patients requires the identification of risk factors for anxiety in patients with epilepsy. Zhong et al. followed up 157 newly diagnosed adult epilepsy patients for 12 months, two important predictors of anxiety disorders were screened from a variety of demographic characteristics such as seizure type, income, and educational background, namely the depression level at baseline and the number of anti-seizure medication (ASM) during follow-up. To a certain extent, it provides a theoretical basis for preventing psychological disorders in patients with epilepsy.

Depressive disorders are less common than anxiety, but not uncommon in patients with temporal lobe epilepsy (TLE). Studies have shown that epilepsy and depression have similar networks in neuropsychiatric disorders, which provides a theoretical basis for the high incidence of epilepsy comorbid depression. Therefore, Sun et al. used EEG microstate analysis for the first time to present the temporal fluctuations of the EEG topography in comorbid depression in patients with TLE and found that TLE patients with the depressive disorder had a shorter microstate time course and more high incidence per second, and compared with the normal group, there was no difference in the coverage of microstate A-D between the two groups, which strongly confirmed alterations in a specific subset of the subsecond functional states of the brain. Chang et al. Using resting-state functional magnetic resonance imaging to observe brain functional connectivity (FC) and degree centrality (DC) in TLE with ictal panic (IP). Compared with TLE without IP and healthy people, TLE patients with IP had significantly higher DC values and increased FC. This opens new doors for further exploration of the neuroimaging mechanisms of IP in TLE patients.

In addition, not only does epilepsy comorbidity bring psychological barriers to patients, but uncontrollable seizures in public places may expose patients to social discrimination. Wu et al. investigated epilepsy stigma attitudes in 310 Chinese native-speakers by using the Simplified Chinese Mandarin version of the Stigma Scale of Epilepsy and subsequently verified the scale's accuracy. To some extent, the degree of disease stigma is quantified, and it provides direction for the development of effective public interventions in the future. Seizures can also be psychologically traumatic for the patient. Using a scale to assess traumatic experienced seizures in patients with pharmacoresistant focal epilepsy, Mariotti et al. found that seizures are the source of the development of postepileptic seizure-posttraumatic stress disorder (PS-PTSD) and noted that early identification and treatment could improve patients' quality of life.

Children with epilepsy are a particularly distinct group of patients with epilepsy. The disease itself will harm children's psychology and adversely affect the quality of life of their families. Operto et al. surveyed 103 children with different forms of epilepsy and 93 in a control group and found that compared with their peers, children with epilepsy were more likely to have emotional and behavioral problems, and the stress of parents in the epilepsy group was significantly higher than that in the control group. Therefore, it is essential to identify emotional and behavioral problems in children with epilepsy early and support their parents accordingly. This is similar to the study by Wei et al. who pointed out that the incidence of depression among primary caregivers of children with epilepsy was close to 70%, and the degree of depression in caregivers was positively correlated with the severity of epilepsy in children; the study by Zhang et al. found that the caregivers' anxiety status, sleep quality, family role dimension, family general function dimension, and the number of co-caregivers were predictors of depression status in caregivers. João et al. assessed anxiety and depression in adults with epilepsy and their caregivers, and although epilepsy patients were more likely than their caregivers to experience depression and suicidal ideation, the proportion and intensity of clinical anxiety symptoms were similar. All three studies point to concern not only for the psychological problems of people with epilepsy but also for their caregivers.

Due to the coronavirus disease 2019 (COVID-19) pandemic, all patients' medical examinations have been delayed, which may worsen patients' diseases. Therefore, Niimi et al. speculated that psychological differences might exist between patients with epilepsy who underwent epilepsy surgery before and during the COVID-19 pandemic, and used the Zung Self-Rating Depression Scale (SDS) to evaluate the difference in depression between the two groups of patients. The results showed that the SDS score was higher in the pre-pandemic group than in the within-pandemic group, indicating that patients with less positive outlooks may be less likely to seek medical attention during periods of societal or personal stress.

Due to the high cost of surgery, some patients still prefer drug therapy. Levetiracetam is considered one of the first-choice drugs for patients with brain tumor-related epilepsy, but side effects come with it. Dono et al. retrospectively analyzed 28 patients with brain tumors and found that prophylactic use of levetiracetam had a higher incidence and severity of psychiatric symptoms than controls. Accurate epileptological evaluations in patients with brain tumors are mandatory to select who would benefit most from ASM.

## Author contributions

ZX, XT, XW, YC, QX, and GA organized this Research Topic and wrote the editorial. All authors have approved the final version of the editorial.

## Acknowledgments

We thank the authors for contributing their valuable work to this Research Topic and the reviewers for their constructive comments. We are also grateful to the editorial board for approving this topic and we hope this issue will advance the state of research on the comorbidities of epilepsy.

## Conflict of interest

The authors declare that the research was conducted in the absence of any commercial or financial relationships that could be construed as a potential conflict of interest.

## Publisher's note

All claims expressed in this article are solely those of the authors and do not necessarily represent those of their affiliated organizations, or those of the publisher, the editors and the reviewers. Any product that may be evaluated in this article, or claim that may be made by its manufacturer, is not guaranteed or endorsed by the publisher.

